# Detailed statistical analysis plan for the pulmonary protection trial

**DOI:** 10.1186/1745-6215-15-510

**Published:** 2014-12-23

**Authors:** Katrine B Buggeskov, Janus C Jakobsen, Niels H Secher, Thomas Jonassen, Lars W Andersen, Daniel A Steinbrüchel, Jørn Wetterslev

**Affiliations:** Department of Cardiothoracic Anaesthesiology, The Heart Centre, Rigshospitalet 4142, Blegdamsvej 9, DK-2100 Copenhagen Denmark; The Copenhagen Trial Unit, Centre for Clinical Intervention Research, Rigshospitalet 7812, Blegdamsvej 9, DK-2100 Copenhagen Denmark; Department of Anaesthesiology, Rigshospitalet 2041, Blegdamsvej 9, DK-2100 Copenhagen Denmark; Department of Biomedical Sciences, The Panum Institute, University of Copenhagen, Blegdamsvej 3, DK-2200 Copenhagen Denmark; Department of Cardiothoracic Surgery, The Heart Centre, Rigshospitalet 2152, Blegdamsvej 9, DK-2100 Copenhagen Denmark

**Keywords:** Cardiac surgery, Chronic obstructive pulmonary disease, Pulmonary dysfunction, Cardiopulmonary Bypass, Pulmonary perfusion, Randomized clinical trial, Statistical analysis plan

## Abstract

**Background:**

Pulmonary dysfunction complicates cardiac surgery that includes cardiopulmonary bypass. The pulmonary protection trial evaluates effect of pulmonary perfusion on pulmonary function in patients suffering from chronic obstructive pulmonary disease. This paper presents the statistical plan for the main publication to avoid risk of outcome reporting bias, selective reporting, and data-driven results as an update to the published design and method for the trial.

**Results:**

The pulmonary protection trial is a randomized, parallel group clinical trial that assesses the effect of pulmonary perfusion with oxygenated blood or Custodiol™ HTK (histidine-tryptophan-ketoglutarate) solution versus no pulmonary perfusion in 90 chronic obstructive pulmonary disease patients. Patients, the statistician, and the conclusion drawers are blinded to intervention allocation. The primary outcome is the oxygenation index from 10 to 15 minutes after the end of cardiopulmonary bypass until 24 hours thereafter. Secondary outcome measures are oral tracheal intubation time, days alive outside the intensive care unit, days alive outside the hospital, and 30- and 90-day mortality, and one or more of the following selected serious adverse events: pneumothorax or pleural effusion requiring drainage, major bleeding, reoperation, severe infection, cerebral event, hyperkaliemia, acute myocardial infarction, cardiac arrhythmia, renal replacement therapy, and readmission for a respiratory-related problem.

**Conclusions:**

The pulmonary protection trial investigates the effect of pulmonary perfusion during cardiopulmonary bypass in chronic obstructive pulmonary disease patients. A preserved oxygenation index following pulmonary perfusion may indicate an effect and inspire to a multicenter confirmatory trial to assess a more clinically relevant outcome.

**Trial registration:**

ClinicalTrials.gov identifier: NCT01614951, registered on 6 June 2012

**Electronic supplementary material:**

The online version of this article (doi:10.1186/1745-6215-15-510) contains supplementary material, which is available to authorized users.

## Update

### Introduction

The pulmonary protection trial (PP-Trial) is a randomized clinical trial that assesses the effect of pulmonary perfusion with oxygenated blood or Custodiol™ HTK (histidine-tryptophan-ketoglutarate) solution in patients suffering from chronic obstructive pulmonary disease (COPD) who undergo cardiopulmonary bypass (CPB)-dependent cardiac surgery [1]. The International Conference on Harmonization of good clinical practice and others recommends that clinical trials should be analyzed according to a pre-specified plan to prevent outcome reporting bias and data-driven analysis results [2–4]. This paper describes the statistical analysis plan for the PP-Trial. Recruitment, randomization, and inclusion of 90 patients were completed by 7 November 2013. All patients were followed for 90 days and the last 90-day follow-up was completed on 3 March 2014. The main publication of the PP-Trial results will adhere to this statistical analysis plan as approved by the steering group. The statistical analysis plan was amended to on the presentation of the trial at ClinicalTrials.gov before data analysis was commenced.

### Trial overview

The PP-Trial is a randomized, parallel group clinical trial comparing pulmonary perfusion with oxygenated blood or Custodiol HTK solution versus no pulmonary perfusion during CPB-dependent cardiac surgery in COPD patients. The patients, the statistician, and conclusion drawers are blinded to intervention allocation. Informed consent was obtained from each patient before inclusion in the trial. The population represents adult patients with COPD receiving coronary artery bypass grafting (CABG), aortic valve replacement (AVR), or the two interventions in combination. The trial background, design, and rationale have been published [1].

The PP-Trial is registered at ClinicalTrials.gov (identifier: NCT01614951) and is carried out in compliance with the Helsinki Declaration and approved by the Committees on Biomedical Research Ethics of The Capital Region of Denmark (approval number: H-1-2012-024), the Danish Medicines Agency (approval number: 2012024017, EudraCT number: 2011-006290-25, protocol code: 4141), and the Danish Data Protection Agency (approval number: 2011-41-7051).

### Sample size

The main comparison will be each of the pulmonary perfusion groups (oxygenated blood or Custodiol HTK solution) compared with no pulmonary perfusion. An exploratory comparison between the two pulmonary perfusion groups will be performed in case the main comparison indicates a benefit of pulmonary perfusion on the primary outcome.

With three groups and thereby multiple comparisons the risk of a type 1 error increases [5]. To limit the family-wise error rate to 0.05, considering two main comparisons and one exploratory comparison, we adjusted the significance level for the primary outcome to α = 0.025 (0.05/2) for each of the three comparisons [6]. With an acceptable risk of type II error of 0.10, we estimated the necessary sample size. The anticipated intervention effects and variance were based on data from previously randomized clinical trials [7, 8]. The sample size calculation was based on an assumed mean difference in the PF (partial pressure of oxygen in arterial blood/fraction of inspired oxygen) ratio of 55 mmHg and a standard deviation (SD) of 50 mmHg for the comparison of pulmonary perfusion with oxygenated blood versus no pulmonary perfusion [7], and an assumed mean difference in the PF ratio of 150 mmHg and a SD of 110 mmHg for the comparison of pulmonary perfusion with Custodiol HTK solution versus no pulmonary perfusion [8]. The sample size estimation showed that we would need 22 patients in two groups to detect or reject a relevant mean difference of 55 mmHg in the PF ratio between pulmonary perfusion with oxygenated blood versus no pulmonary perfusion. It also showed that we would need 15 patients in two groups to detect or reject a relevant mean difference of 150 mmHg in the PF ratio between pulmonary perfusion with Custodiol HTK solution versus no pulmonary perfusion. We therefore estimated that we would need three groups of 30 patients to preserve power in the complete case analysis in case of eventual patient dropouts or loss to follow-up. If we find a positive effect on the oxygenation index for both of the pulmonary perfusion groups when compared with patients who do not receive pulmonary perfusion, the exploratory comparison of the two pulmonary perfusion groups becomes interesting. To calculate a hypothetical sample size for this comparison we set a value for the anticipated mean difference and SD to 30 mmHg and 30 mmHg, respectively. With a significance level for the primary outcome still being α = 0.025 and the power set to 90%, we calculated the sample size for this comparison to be 26 patients in each group.

### Stratification and design variables

We will use preoperative lung function as a stratification variable. Preoperative lung function will be divided in to two groups: 1) mild COPD, and 2) moderate, severe, or very severe COPD. Predefined design variables will be: age, forced expiratory volume in 1 second (FEV1), left ventricular ejection fraction, and the patient’s baseline oxygenation index measured after anaesthetic induction.

### Definition of the outcome measures

The outcomes will be defined as primary, secondary, and exploratory. Only results on the primary and secondary outcome measures will be reported in the first published report of the PP-Trial. The exploratory outcome measures will be reported in a separate publication(s).

### Primary outcome

The primary outcome will be the oxygenation index measured six times from anaesthetic induction, through surgery, and until 24 hours after anaesthetic induction, of which the first is considered a baseline measurement and used as a covariate.

### Secondary outcome measures including serious adverse events

The secondary outcomes measures will be: 1) oral tracheal intubation time (hours) after primary surgery, 2) days alive outside the intensive care unit within a follow-up of 90 days, 3) days alive outside the hospital within a follow-up of 90 days, 4) 30- and 90-day mortality, and 5) patients with one or more of the following selected serious adverse events: pneumothorax or pleural effusion requiring drainage, major bleeding, reoperation, severe infection, cerebral event, hyperkaliemia, acute myocardial infarction, cardiac arrhythmia, renal replacement therapy, and readmission with a respiratory-related problem (such as pneumonia or acute exacerbation of COPD). A list of the selected serious adverse events is displayed in Table 1.

**Table 1:** **Serious adverse events reported during the trial**

Serious adverse events	Definition
Pneumothorax or pleural effusion	Requiring drainage
Major bleeding	More than 700 mL within the first 24 hours after surgery.
Reoperation	Cardiac ischemia, server hemorrhage, cardiac tamponade, or pericardial effusion requiring reoperation.
Severe infection	Sepsis, septic shock, and other serious infections.
Cerebral event	Transient cerebral ischemic attack or stroke, myoclonic and/or tonic-clonic seizures.
Hyperkaliemia	Hyperkaliemia (>5.5 mmol/L and treated with medicine).
Acute myocardial infarction	ST- or non-ST-elevated myocardial infarction
Cardiac arrhythmia	Atrial fibrillation, atrial flutter, ventricular tachycardia, ventricular fibrillation, and cardiac arrest mandating cardiopulmonary resuscitation.
Renal replacement therapy	Continuous or intermittent
Readmission with a respiratory-related problem	For example, pneumonia or acute exacerbation of chronic obstructive pulmonary disease.

### Exploratory outcome measures

The exploratory outcome measures will be: 1) tracheostomy, 2) difference in thoracic electrical admittance in reflection of accumulation of extra-vascular lung water during surgery and within 24 hours, 3) difference in cell and differential counts in bronchoalveolar lavage fluid, 4) difference in plasma markers of inflammation (such as interleukin 6 and monocyte chemotactic protein 1), 5) difference in alveolar membrane thickness and decrease in surface area to indicate intracellular fluid accumulation, and 6) difference between the preoperative pulmonary function test and the one performed 90 days after surgery.

### Baseline characteristics

The baseline characteristics will be assessed from inclusion in the trial to after anaesthetic induction. The baseline characteristics will be:

- Demographic characteristics:
- Age
- Gender
- Comorbidity (reported if the frequency is above or equal to 10% (three patients per group) in any of the intervention groups; COPD will be reported regardless of its frequency):
- Self-reported COPD
- Pulmonary hypertension
- Arterial hypertension
- Chronic atrial fibrillation or flutter
- Chronic heart failure (New York Heart association Class III or worse)
- American Society of Anesthesiologists (ASA) class VI or worse
- Left ventricular ejection fraction
- Recent (within three months of surgery) acute myocardial infarction 2.i. Insulin-dependent diabetes mellitus
- Renal function (estimated creatinine clearance calculated by the Cockcroft-Gault formula)
- Previous transient cerebral ischemic attack or stroke
- Previous percutaneous coronary intervention
- Implantable cardioverter-defibrillator and/or pacemaker
- Alcohol consumption >14 units for women or >21 units for men per week
- Tobacco pack years
- Pre-surgery pulmonary data:
- Percent predicted FEV1
- Global Initiative for Chronic Obstructive Lung Disease (GOLD) classification stage II or worse.
- Surgical data:
- Urgent and/or elective surgery
- Type of surgery (CABG, AVR or CABG and AVR).

### Population and handling of missing data

The primary conclusion of the trial will be based on the results of the primary outcome. If the result of the primary outcome is not statistically significant, the conclusion will be that there is no significant difference between the interventions. The results on all other types of outcomes will be reported for hypothesis-generating purposes. However, we will inspect the confidence interval (CI) to asses if the CI for the group difference contains values of importance, so that we cannot rule out interesting differences.

The primary analysis will include a modified intention-to-treat population, which is defined as all randomized patients, except patients who did not receive CPB-dependent cardiac surgery [9]. A secondary analysis will include the per-protocol population excluding patients with major protocol violations defined as: 1) patients who were randomized to an intervention but did not receive any intervention; and 2) patients who received an incorrect intervention. The dependent variable will be the oxygenation index measured six times and the covariates will be the stratification (mild COPD or moderate, severe, or very severe COPD), design (age, FEV1, left ventricular ejection fraction, and the patient’s baseline oxygenation index measured after anaesthetic induction) and intervention-group variable.

For the modified intension-to-treat population and the per-protocol population two analyses will be performed for primary and secondary outcomes. The first analysis will be on the patients who met the inclusion criteria but did not meet the exclusion criteria, with adjustment for the stratification variable. The second analysis will be an analysis adjusted for both the stratification variable and the design variables [10]. The conclusions of the trial will be based on the primary analysis.

Linear mixed-effects model analysis of longitudinal data will be used to analyze the primary outcome. The linear mixed-effects model approach handles missing data appropriately and we will therefore not use multiple imputation or other methods to handle missing data in regard to the primary outcome [11]. If data missingness for the remaining outcomes represent less than 5% (five patients), a complete case analysis without input of missing values will be performed. If missing data are more than 5%, a blinded statistician will assess whether missing data are completely at random and may be ruled out based on a rational assessment of the pattern of missing data [12], and Little’s test will be used if doubt remains [13]. If it is concluded that missing data are not completely at random, multiple imputation using chained equations [14, 15] will be performed by creating 10 input data sets under the assumption of that the missing data are at random [16, 17]. We will use the stratification-, design-, and intervention-variables, as well as other known predictive outcomes, in the multiple imputation to estimate the missing values. If multiple imputation is used, then the primary result of the trial will be based on these data. The unadjusted, non-imputed analysis will also be made available. If multiple imputation is used, we use a best-worst worst-best case scenario as a supplement and a form of sensitivity analysis to assess the potential range of impact of the missing data for the trial results. In the ‘best-worst’ case scenario, it is assumed that all patients lost to follow-up in the experimental group have had a beneficial outcome (have survived, had no serious adverse events, and so forth), and all those with missing outcomes in the control group have had a harmful outcome (have not survived, have had a serious adverse event, and so forth). Conversely, in the ‘worst-best’ case scenario, it is assumed that all patients who were lost to follow-up in the experimental group have had a harmful outcome, and that all those lost to follow-up in the control group have had a beneficial outcome. When continuous outcomes are used, a ‘beneficial outcome’ will be defined as the group mean plus two standard deviation (SD) of the group mean, and a ‘harmful outcome’ will be defined as the group mean minus two SD of the group mean.

We will assess the validity of the trial results according to the five-point procedure as suggested by Jakobsen *et al*. [5]. This procedure will include the mentioned adjustments of thresholds for significance according to the number of primary outcome comparisons. We will use a *P* value threshold for significance of 0.025 (0.05/2 because two primary outcome comparisons are used) and a Bayes factor threshold for significance of 0.1 [5].

### Statistical analysis

#### Trial profile

**Figure 1:**
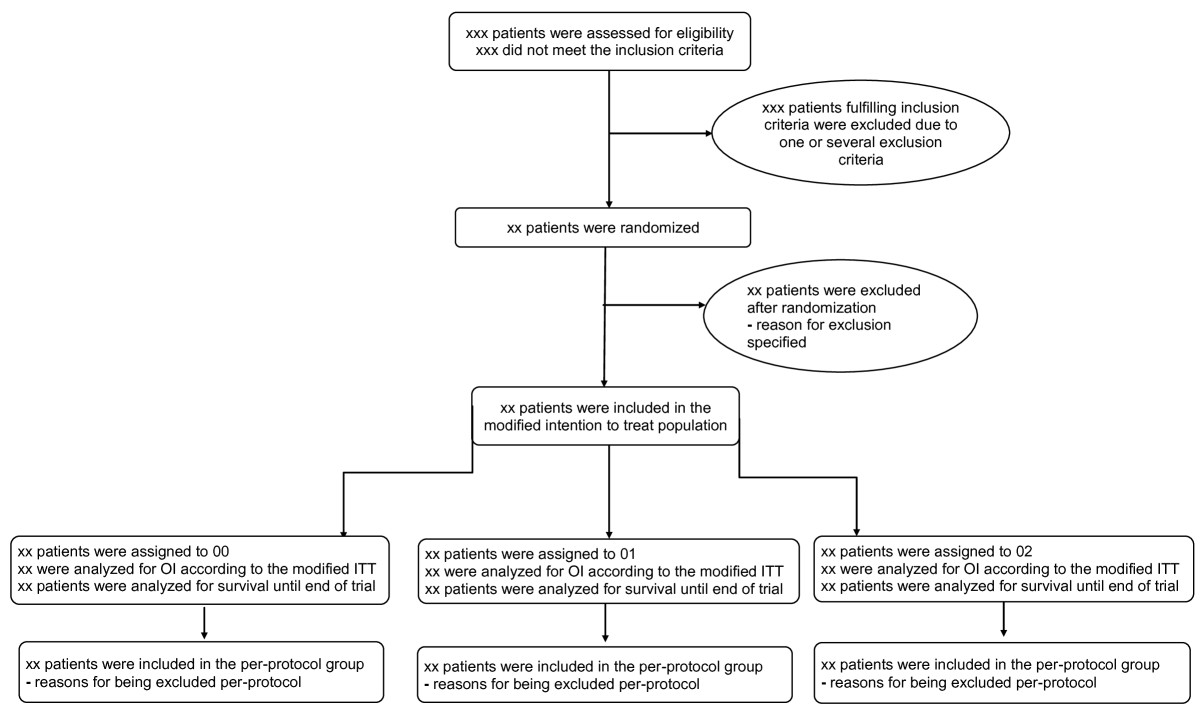
**CONSORT flow diagram.** ITT, intension-to-treat; OI, oxygenation index.

The flow of trial patients will be displayed in a Consolidated Standards of Reporting Trials (CONSORT) diagram (see Figure 1) [18]. The number of screened patients who fulfilled trial inclusion criteria, and the number included in the primary and secondary analyses, as well as all reasons for exclusions in primary and secondary analyses will be reported.

### Statistical analysis of the primary outcome

The differences between the groups on the primary outcome will be tested with a mixed-effects model with time as a continuous variable. The oxygenation index is for all patients measured at six time points: (1) after anaesthetic induction, (2) 10 to 15 minutes after CPB, (3) 120 to 125 min after CPB, (4) 240 to 245 min after CPB, (5) 360 to 365 min after CPB, and (6) 24 hours post-anaesthetic induction. The baseline measurement for the primary outcome is the oxygenation index measured after anaesthetic induction. This measurement will be included in the analysis as a covariate.

Since the time points for measurement of the oxygenation index do not have equidistant time intervals, we will also carry out a sensitivity analysis with time as an ordinal variable where the time intervals are equal. The mixed-effects model results will be presented as *P* values and 95% CI.

In the mixed-effects model we will use both an ‘unstructured' and a '1. order autoregressive' covariance matrix and choose the matrix resulting in the lowest Bayesian information criterion. To assess, if the underlying assumptions behind the mixed-effects model analysis are fulfilled, we will investigate normal quantile plots of residuals, standardized residuals, and random effects. If the underlying assumptions behind the mixed-effects model analysis are clearly violated then we will use a generalized estimation equation for the analysis.

Secondly we will use analysis of covariance (ANCOVA) to compare the three groups at the 24 hours after anaesthetic induction time point. The baseline oxygenation index will also be included here in the analysis as a covariate. At this time point the oxygenation index is among others used to evaluate if the patients’ circulatory and respiratory system is stable enough to discharge them from the intensive care unit to the ward. To assess whether the assumptions for the ANCOVA are fulfilled, we will investigate normal quantile plots of residuals and residuals versus fitted values. ANCOVA results will be presented as *P* values, 95% CI, and unadjusted mean differences.

### Statistical analysis of the secondary outcome measures including serious adverse events

Proportions of patients with one or more serious adverse events, and 30 and 90 days mortality will be analyzed as a dichotomous variable using logistic regression and results will be presented as relative risks, 95% CI, and exact *P* values. We will also produce a table showing how many patients in each group had one, two, three, four, and so forth adverse events, respectively. We will test the underlying assumptions for the logistic regression by plotting Pearson residuals against both fitted values and against each continuous variable.

All other secondary outcomes will be analyzed as count variables using the van Elteren test [19]. Count data results will be presented as mean and median differences using bootstrapping to show 95% CI. The van Elteren test is non-parametric and it is therefore not necessary to test for underlying assumptions. For the secondary outcomes we will calculate the *P* value and divide the statistical significance into three groups: 1) *P* >0.05: not statistically significant; 2) *P* = 0.01 to 0.05: dubious statistically significance; and 3) *P* <0.01: statistically significant.

### Characteristics of patients with baseline comparisons

We will present the description of baseline characteristics by intervention group. Discrete variables will be summarized by frequencies and percentages calculated according to the number of patients for whom data are available. Where values are missing, the actual denominator will be stated. Continuous variables will be summarized using standard measures of central tendency and dispersion, using either mean ± SD for data with normal distribution, or median and interquartile range for non-normally distributed data. Tests for interaction between the intervention and each stratification and design variables used to identify subgroups will be exploratory.

### Outline of figures and tables

The first figure will be a CONSORT flow chart as specified in Figure 1. The second figure will be an oxygenation index graph for the three groups with hours 0 to 24 on the x-axis and the mean oxygenation index on the y-axis. Data for the second figure will be the complete cases, and therefore there is an underlying assumption that the data is missing completely at random; interpretation of the figure should therefore be with precaution. The third figure will be a Kaplan-Meier plot of survival in the three groups during the total trial period (15 months). The fourth figure will be a forest plot of intervention effects stratified for the subgroups defined by the stratification (mild COPD or moderate, severe, or very severe COPD) and design variables: age dichotomized around the median, FEV1 dichotomized around the median, left ventricular ejection fraction dichotomized around the median, and the patient’s baseline oxygenation index dichotomized around the median.

The first table will be the baseline characteristics of the modified intention-to-treat population, the second table will be the prevalence of the binary and continuous outcome measures adjusted for the stratification variable, and the third table will be the results of the adjusted (stratification and design variables) complete case and multiple imputation analyses, with a 95% CI and exact *P* values in tabular form. A fourth table showing how many patients in each group had one, two, three, four, and so forth adverse events will also be produced.

### Deviations from the initial design and method of the trial

Due to a much lower than expected number of patients receiving transcatheter aortic-valve implantation, we were forced to exclude that intervention as our planned secondary control group and that comparison is therefore deleted in this statistically analysis plan.

Some of the secondary outcome measures are not completed due to lack in financial support and collaborates not able to fulfill assignments. These are as follows: number and degree of activated alveolar macrophages and T-cells measurement and differentiation in bronchoalveolar lavage fluid, degree of uncoupling the oxidative phosphorylation in the musculus transversus abdominis, and pulmonary tissue mitochondria.

## Discussion

This paper presents the detailed statistical analysis plan for the PP-Trial in order to avoid risks of outcome reporting bias and data-driven results. Of the pre-specified results from the trial, we plan to report the primary and secondary outcome measures in the main publication. Due to the complexity of the CPB-induced inflammatory response, considered an exploratory outcome, we have scheduled separate publication(s).

We will use a pragmatic adjustment for multiplicity described in the following. The primary conclusion of the trial will be based on the result of the primary outcome and if this result lacks statistically significance, the overall conclusion will be that there is no significant difference between the compared interventions. We will analyze data in accordance to the modified intention-to-treat principle and, if necessary, use data sets generated by multiple imputations, and a best-worst/worst-best case scenario to assess the potential impact of the missing data on the results.

### Strengths

Our methodological strength is the predefined methodology of the design and statistical analysis plan [3]. Also, we take the multiplicity problem into account and use validated analytical methods, including systematic tests, for underlying assumptions. We strengthen our results by use of multiple imputations for missing data and the best-worst/worst-best case scenario to show range of the results’ uncertainty.

### Limitations

The statistical analysis plan has limitations. The results on the primary outcome are analyzed by a linear mixed-effects model which can be difficult to interpret with analytical complexity and may be difficult to test for underlying assumption. Further, the sample size estimation was based on a slightly different outcome measure, the PF ratio (partial pressure of oxygen in arterial blood/fraction of inspired oxygen), which does not include airway pressure as for the oxygenation index, and the estimated sample size does not necessarily comply with what is needed in the mixed-effects model. For some of the oxygenation index measurements the patient will be extubated breathing atmospheric air or receiving oxygen supply by a nasal catheter or oxygen mask. In these cases, the oxygenation index is calculated by first converting the external oxygen supply to an estimated fraction of inspired oxygen [20], and second by setting the mean airway pressure to 1, removing that parameter from the equation. That leaves us with comparisons of the oxygenation index under different circumstances (intubated or extubated), and calculations which may constitute a source of error in our results.

We use a number of surrogate outcome measures with questionable clinical relevance, and even though we have classified the trial as a pilot trial, there is a risk that the trial via valid statistical methodology will indicate that the assessed interventions are beneficial, which could be misleading because of the unestablished clinical relevance of the surrogate outcome measures. Further, we have included a number of clinically relevant secondary outcome measures but we are likely not to have sufficient power to reliable access and conclude on these measures. Finally, we use multiple imputation to handle missing data on the remaining outcome measures and it cannot be ruled out that data missing are not at random and multiple imputation may produce biased results.

## Conclusions

This article describes the principles of statistical analyses used in the PP-Trial for the primary publication of the main outcome measures in order to minimize risk of data-driven results and outcome reporting bias.

## Authors’ original submitted files for images

Below are the links to the authors’ original submitted files for images. Authors’ original file for figure 1

## Abbreviations

ANCOVA: Analysis of covariance
ASA: American Society of Anesthesiologists
AVR: Aortic valve replacement
CABG: Coronary artery bypass graft
CI: Confidence interval
CONSORT: Consolidated standards of reporting trials
COPD: Chronic obstructive pulmonary disease
CPB: Cardiopulmonary bypass
FEV1: Forced expiratory volume in 1 second
GOLD: Global initiative for chronic obstructive lung disease
HTK: Histidine-tryptophan-ketoglutarate
PP-Trial: Pulmonary protection trial
SD: Standard deviation.
