# Supplementary material for: Detailed statistical analysis plan for the pulmonary protection trial
Source: Trials. 2014 Dec 23;15:510. doi: 10.1186/1745-6215-15-510 (PMC4307213; doi:10.1186/1745-6215-15-510)
Supplement: Supplementary file 1 — Authors’ original file for figure 1 [file 13063_2014_2387_MOESM1_ESM.pdf]

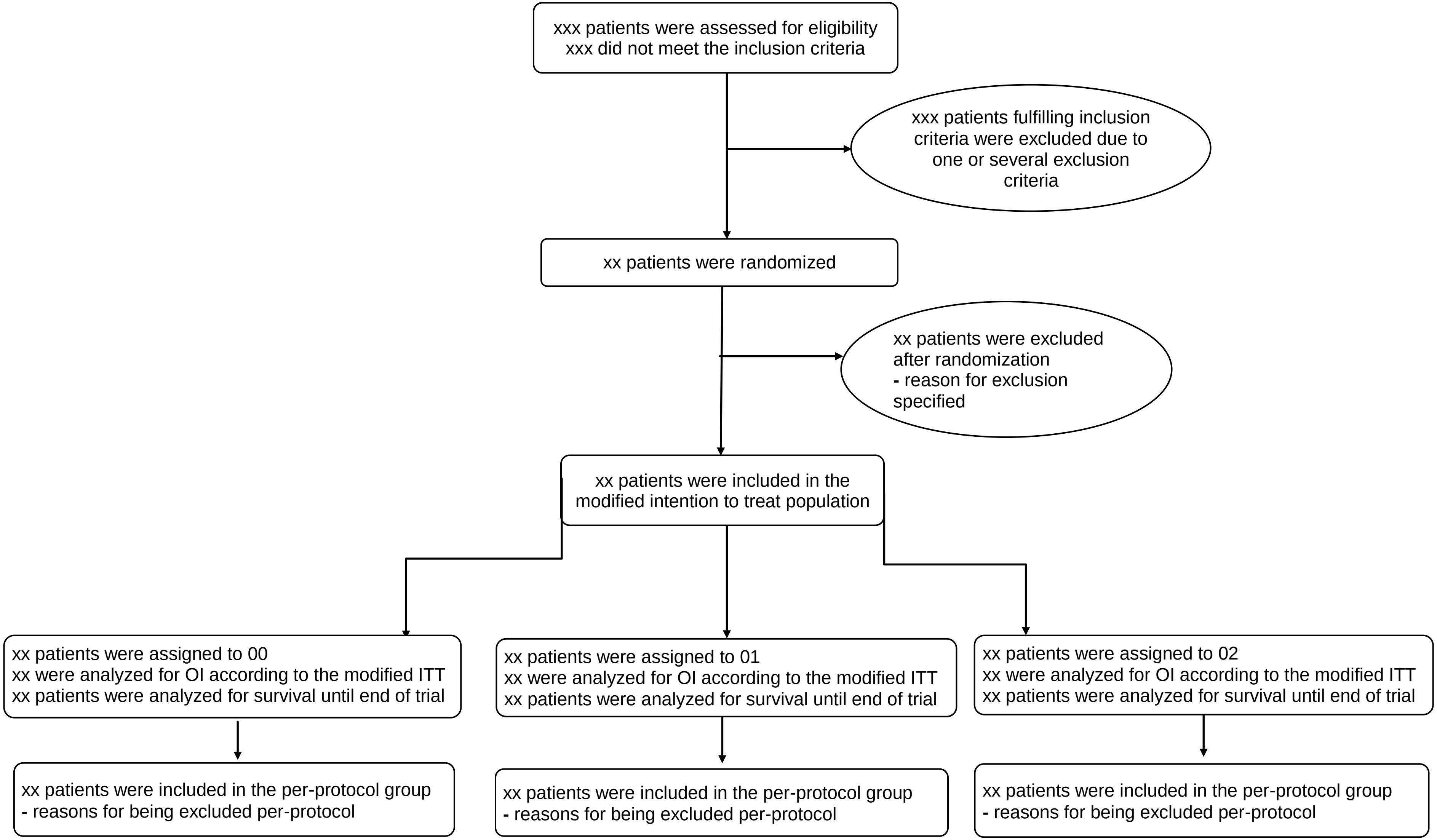
xxx patients were assessed for eligibility
xxx did not meet the inclusion criteria
xxx patients fulfilling inclusion criteria were excluded due to one or several exclusion criteria
xx patients were randomized
xx patients were excluded after randomization
- reason for exclusion specified
xx patients were included in the modified intention to treat population
xx patients were assigned to 00
xx were analyzed for OI according to the modified ITT
xx patients were analyzed for survival until end of trial
xx patients were assigned to 01
xx were analyzed for OI according to the modified ITT
xx patients were analyzed for survival until end of trial
xx patients were assigned to 02
xx were analyzed for OI according to the modified ITT
xx patients were analyzed for survival until end of trial
xx patients were included in the per-protocol group
- reasons for being excluded per-protocol
xx patients were included in the per-protocol group
- reasons for being excluded per-protocol
xx patients were included in the per-protocol group
- reasons for being excluded per-protocol
